# NaringeninーA potential nephroprotective agent for diabetic kidney disease: A comprehensive review of scientific evidence

**DOI:** 10.17305/bb.2024.10511

**Published:** 2024-12-01

**Authors:** Estefania Valle-Velázquez, Oscar René Zambrano-Vásquez, Fernando Cortés-Camacho, Laura Gabriela Sánchez-Lozada, Gustavo Guevara-Balcázar, Horacio Osorio-Alonso

**Affiliations:** 1 Departamento de Fisiopatología Cardio-Renal, Instituto Nacional de Cardiología Ignacio Chávez, Mexico City, Mexico; 2 Sección de Estudios de Posgrado e Investigación, Escuela Superior de Medicina, Instituto Politécnico Nacional, Mexico City, Mexico

**Keywords:** Diabetes, chronic kidney disease (CKD), naringenin, oxidative stress, hypertension, inflammation

## Abstract

Diabetes mellitus (DM) is a chronic disease characterized by persistent hyperglycemia, which is a major contributing factor to chronic kidney disease (CKD), end-stage renal disease (ESRD), and cardiovascular-related deaths. There are several mechanisms leading to kidney injury, with hyperglycemia well known to stimulate oxidative stress, inflammation, tissue remodeling, and dysfunction in the vascular system and organs. Increased reactive oxygen species (ROS) decrease the bioavailability of vasodilators while increasing vasoconstrictors, resulting in an imbalance in vascular tone and the development of hypertension. Treatments for diabetes focus on controlling blood glucose levels, but due to the complexity of the disease, multiple drugs are often required to successfully delay the development of microvascular complications, including CKD. In this context, naringenin, a flavonoid found in citrus fruits, has demonstrated anti-inflammatory, anti-fibrotic, and antioxidant effects, suggesting its potential to protect the kidney from the deleterious effects of diabetes. This review aims to summarize the scientific evidence of the effects of naringenin as a potential therapeutic option for diabetes-induced CKD.

## Introduction

Diabetes mellitus (DM) is a metabolic disease characterized by hyperglycemia, resulting from defects in insulin secretion or action. This leads to polyuria, polydipsia, polyphagia, and body weight loss. The prevalence of diabetes has reached epidemic proportions, as it is estimated to affect more than 9% of the total world population (more than 463 million people) and is predicted to increase to over 638 million by 2045 [1].

Diabetes management represents a high economic burden, mainly due to the need for glycemic control. Poor control of glycemia leads to damage to target organs and systems, resulting in disability and premature death. The main organs and systems affected by long-term hyperglycemia include the heart, blood vessels, eyes, nerves, and kidneys [2, 3]. In this sense, diabetes is the leading cause of chronic kidney disease (CKD) and end-stage renal disease (ESRD) worldwide [2, 4].

CKD is a non-communicable disease that has become an emergent health problem affecting more than 10% of the global population, (over 800 million people) and significantly contributing to morbidity and mortality rates worldwide [4, 5]. In people with diabetes, the prevalence of CKD has been reported to be between 25% and 38%, with predictions that more than 40% will develop CKD at any stage. Importantly a significant number will develop ESRD and require dialysis and/or kidney transplant [1, 6].

Furthermore, the progression of CKD is accelerated by the coexistence of comorbidities, such as diabetes (1:3) and high blood pressure (1:5) [7]. The coexistence of diabetes and hypertension increases the risk of developing micro- and macrovascular complications, such as CKD and cardiovascular disease (CVD) [8, 9]. In fact, hypertension is prevalent in patients with diabetic nephropathy and increases as renal function declines. Therefore, therapeutic strategies for diabetes must focus on controlling hyperglycemia, oxidative stress, inflammation, and hypertension to retard the progression of CKD [8–10]. In addition, diabetes is commonly associated with other comorbidities, such as dyslipidemia and obesity (features of metabolic syndrome), which themselves represent risk factors for the development of microvascular complications such as CKD [11].

CKD is stratified according to proteinuria, glomerular filtration rate (GFR), and albumin/creatinine ratio (ACR) [12]. In diabetes, CKD (diabetic nephropathy) is characterized by glomerular hyperfiltration, hypertrophy, albuminuria (>300 mg/day), thickening of the basement membrane, mesangial expansion, nodular sclerosis, and tubulointerstitial fibrosis, eventually leading to a progressive decline in GFR, and ultimately, ESRD, in which dialysis or renal replacement therapy to sustain life is needed [13, 14].

### Pathogenesis of diabetes-induced chronic kidney disease

The progression of CKD is multifactorial and results from the complex interaction of several processes, including altered homeostasis, metabolic disorders, hemodynamic abnormalities, increased generation of reactive oxygen species (ROS), proinflammatory mechanisms, and activation of the rennin–angiotensin–aldosterone system (RAAS) [3].

**Figure 1.**
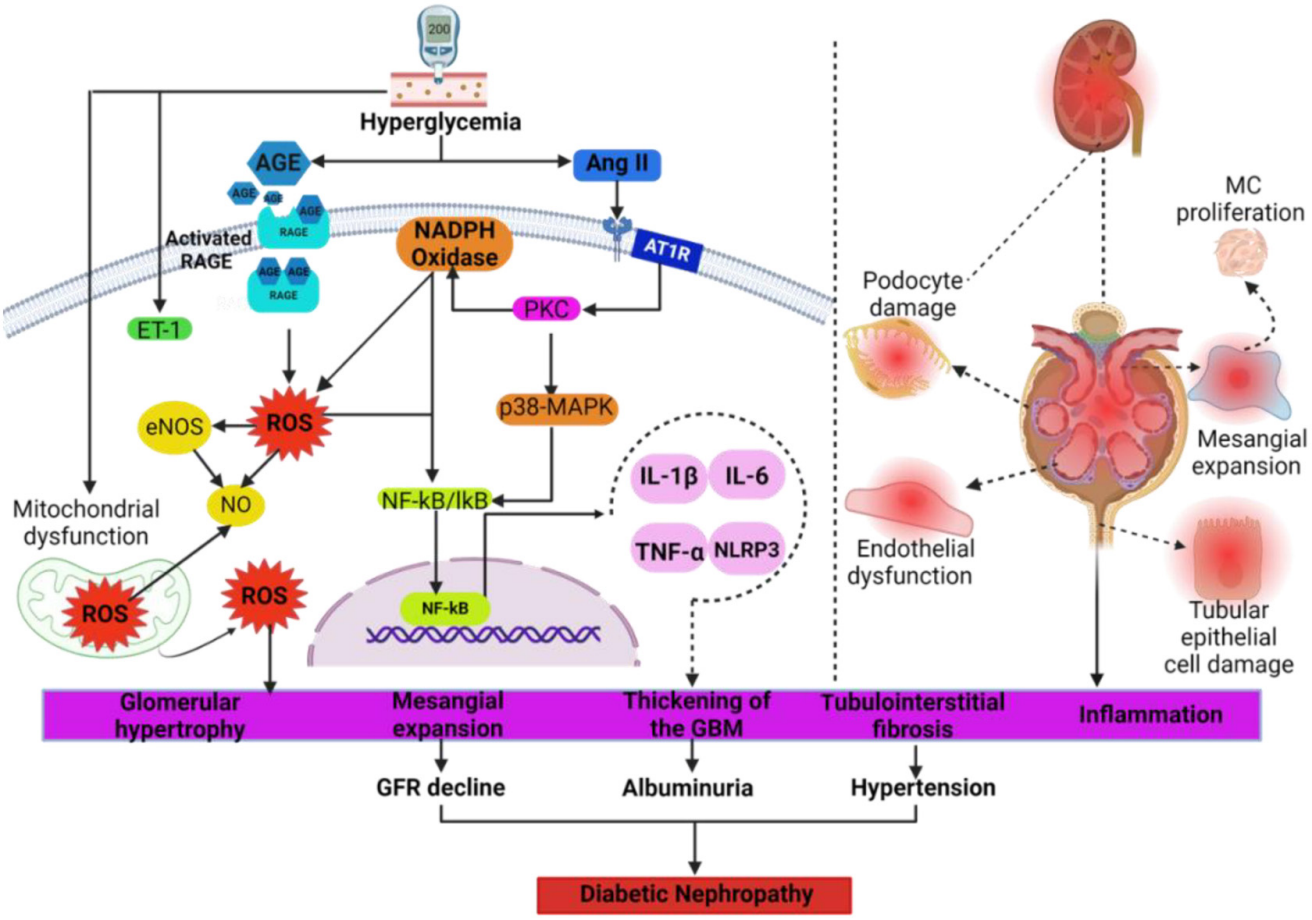
**Hyperglycemia is the major pathogenic mechanism leading to the activation of cellular mechanisms and signaling pathways, which subsequently result in diabetic nephropathy.** Hyperglycemia increases the formation of advanced glycation end-products, and reactive oxygen species, and it activates the renin–angiotensin system, at both systemic and local levels. These pathogenic factors stimulate inflammation and fibrosis in endothelial, glomerular, mesangial, and tubular cells, leading to impaired renal function and ultimately to diabetic nephropathy. AGE: Advanced glycation end products; Ang II: Angiotensin II; AT1R: Angiotensin II type 1 receptor; eNOS: Endothelial NO synthase; ET-1: Endothelin-1; GBM: Glomerular basement membrane; GFR: Glomerular filtration rate; IL-1β: Interleukin 1-beta; IL-6: Interleukin-6; p38-MAPK: P38 mitogen-activated protein kinase; MC: Mesangial cell; NO: Nitric oxide; NLRP3; Nucleotide-binding and oligomerization domain-like receptors; NF-κB: Nuclear factor kappa B; RAGE: Receptor for AGEs; ROS: Reactive oxygen species; TNF-α: Tumor necrosis factor-alpha.

Hyperglycemia triggers mechanisms, such as activation of the polyol pathway flux and the formation of advanced glycation end products (AGEs), which bind to the receptor for AGE (RAGE). This leads to the formation of the AGE–RAGE complex, which activates NADPH oxidase (NOX) and stimulates ROS production (Figure 1). The increase in local angiotensin II (Ang II) synthesis mediates other signaling pathways to produce ROS, such as the protein kinase C/NOX (PKC/NOX) pathway (Figure 1) [15].

Oxidative stress is recognized as a primary factor contributing to the onset of endothelial dysfunction and hypertension in diabetes. Moreover, chronic hyperglycemia induces changes in the vascular endothelium, which plays crucial roles in blood regulation, tissue oxygenation, and, notably, the modulation of vascular tone through the secretion of vasoactive substances at systemic and tissue levels. In this context, diabetes and hypertension activate signaling pathways that stimulate inflammatory processes and oxidative stress, deteriorating endothelial function (Figure 1) [16]. On the other hand, in patients and animals with diabetes nitric oxide (NO)-dependent vasodilation is impaired due to decreased activity or expression of endothelial NO synthase (eNOS), which is caused by oxidative stress and inflammation [10, 17]. In addition, increased ROS leads to the rapid oxidation of NO, reducing its bioavailability and resulting in the predominance of vasoconstrictor substances, such as Ang II and endothelin 1 (ET-1) in endothelial cells [18].

The kidney’s endothelium performs unique functions, including blood filtration at the glomeruli. Peritubular capillaries contribute to the reabsorption, secretion, and elimination of waste products carried out by proximal tubules. Hyperglycemia induces endothelial dysfunction in the diverse renal vascular beds, thus disturbing kidney function [19].

The kidney has all the components of the RAAS, but contrary to the systemic RAAS, it is activated by hyperglycemia [10, 20, 21]. A glucose-response element in the angiotensinogen (Agt) gene promoter mediates the stimulation of intrarenal Agt synthesis by high glucose [22]. Also, renin, a key component in RAAS, is overexpressed under hyperglycemia conditions [23]. Ang II has relevant hemodynamic effects, and its overactivation plays a key role in the development of glomerular hyperfiltration. Increasing AT1 receptor signaling is a determinant in inducing Ang II renal effects [10, 24]. Besides hemodynamic alterations, Ang II stimulates the expression of proinflammatory and profibrotic mediators and activates NOX, stimulating renal production of ROS. This, combined with an increase in transforming growth factor-beta (TGF-β), leads to remodeling of the extracellular matrix in the mesangium and promotes fibrotic processes in the renal tubular interstitium [25].

In the kidney, oxidative stress causes damage to mesangial cells, endothelial cells, and podocytes, impairing the glomerular filtration barrier. This leads to proteinuria and tubulointerstitial fibrosis [26, 27].

Moreover, oxidative stress promotes the aggregation of lymphocytes, neutrophils, and macrophages, which synthesize proinflammatory cytokines, chemokines, growth factors, and transcription factors, aggravating inflammation and oxidative stress [15]. During diabetes and in response to oxidative stress, kidney cells produce proinflammatory substances facilitating the innate immune response through the release of chemokines, adhesion molecules (CAMs), and damage-associated molecular patterns (DAMPs). This increases renal inflammation and promotes the infiltration of neutrophils and macrophages [26]. Part of the inflammatory response in the kidney is mediated by nuclear factor kappa B (NF-κB), which promotes the synthesis of interleukin-1β (IL-1β) and IL-18 [27, 28]. Likewise, increments in serum levels of IL-6 and IL-18 are related to albuminuria, thickening of the glomerular basement membrane (GBM), and increased concentrations of interferon-γ (IFN-γ), IL-1β, and tumor necrosis factor-alpha (TNF-α) (Figure 1) [26, 27]. Further, in patients and experimental models of diabetic nephropathy, the increase in TNF-α, IL-6, and IL-18 in glomerular and proximal tubule cells correlates with microalbuminuria [27, 28].

### Treatments for diabetes-induced chronic kidney disease

The treatment of diabetic nephropathy is focused on delaying or halting the progression of the disease to advanced stages. In both patients and experimental models, it has been demonstrated that controlling hyperglycemia and blood pressure reduces proteinuria, hyperfiltration, and glomerular lesions [7]. However, because diabetes complications are primarily associated with vascular complications, protecting systemic and renal vascular function is also important. Therefore, treatments, in addition to being tailored for controlling hyperglycemia and blood pressure, should include protecting endothelial and renal function and regulating oxidative stress and inflammation.

Managing diabetic nephropathy requires a multidimensional approach encompassing lifestyle adjustments, patient education, and pharmaceutical intervention. Over the years, various medications have been formulated, each targeting distinct mechanisms to safeguard the kidneys, impede disease advancement, and alleviate cardiovascular complications. Recent advancements have introduced novel medication classes, offering substantial potential in combating diabetic nephropathy and its associated cardiovascular risks. However, despite the availability of these drugs, effectively managing this disease continues to pose challenges, with a notable residual risk persisting despite adherence to optimal medical regimens [29]. This is especially true for the risk of developing macro- and microvascular complications [30]. In this context, medicine based on drugs combined with lifestyle changes has increased life expectancy and is considered complementary medicine [31, 32]. In turn, lifestyle changes can include physical activity, as well as improvement in dietary habits, and even the use of traditional medicine, including herbs, fruits, vegetables, and spices. The therapeutic efficacy of complementary medicine is due to the content of active substances that exert beneficial effects on health, known as nutraceuticals [33, 34]. Therefore, studying herbal medicine to find potential compounds that support the conventional or complementary medicine currently used to treat/manage diabetes is justified.

Compounds derived from plants, fruits, or vegetables include a wide group of substances and have demonstrated benefits in reducing the progression of chronic diseases, such as diabetes, hypertension, and CKD [30, 32, 34, 35]. Within these substances are flavonoids, a group of compounds that include several subclasses, such as flavanols, flavanones, isoflavones, anthocyanins, etc. [36, 37].

**Figure 2.**
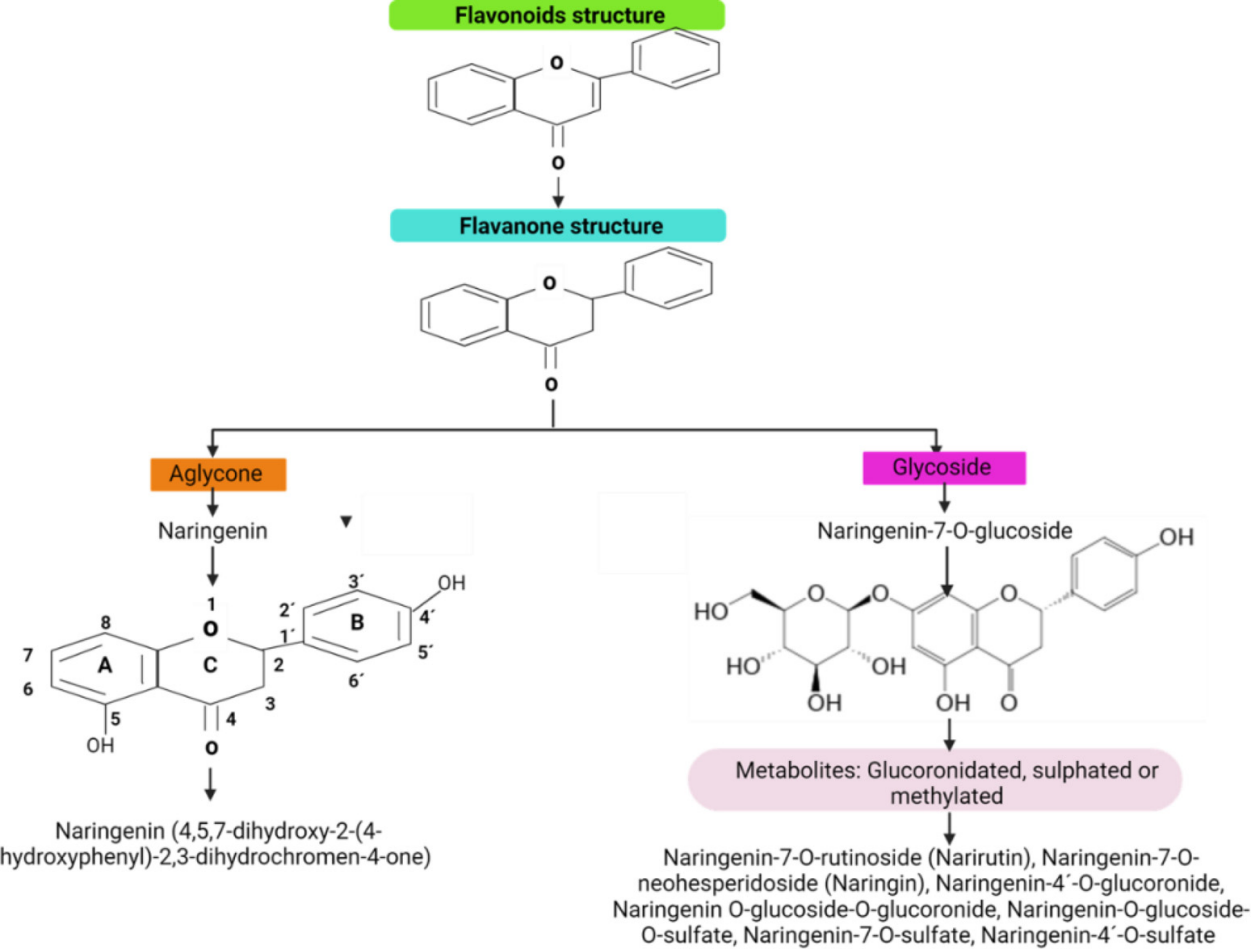
**Flavonoids are a large group of compounds subclassified according to the radical or group attached to their main structure.** Among these subclassifications are flavanones, such as naringenin, which can be found in both aglycone and glycosylated forms. Naringenin and its glycoside forms undergo metabolism through glucuronidation, sulfation, or methylation, resulting in naringenin derivatives.

## Flavonoids

Flavonoids are a large group of compounds present in vegetables, fruits, seeds, grains, and spices. The content of these compounds may vary in leaves, bark, fruit, flower, and stem. The chemical structure of flavonoids is formed by two aromatic rings linked by a carbon chain that forms an oxygenated heterocyclic ring (C6-C3-C6) (Figure 2) [38]. According to certain characteristics, such as the presence of radicals, oxidation, unsaturation degree, or functional groups in their structure, flavonoids are classified as anthoxanthins, flavanones, flavanonols, flavans, chalcones, anthocyanidins, and isoflavonoids [39]. It has been suggested that the antioxidant activity of flavonoids is closely related to the hydroxyl groups in their structure, which confer the capacity for free radical scavenging and metal ion chelating activities (Figure 2) [39]. Flavonoids have been utilized in traditional medicine because this broad group of substances has shown health-promoting activities, such as anti-inflammatory, antioxidative, anti-carcinogenic, anti-hyperlipidemic, and anti-diabetic effects. Numerous flavonoids have undergone evaluation via both in vitro and in vivo experiments, with consistently reproducible effects. This substantiates their utilization as therapeutic options and has facilitated their commercialization in various forms, including tablets, capsules, powder, granules, suspension, or emulsion. Examples of drugs derived from flavonoids include diosmin (3′,5,7-trihydroxy-4′-methoxyflavone 7-rutinoside), hesperidin (3′,5,7-trihydroxy-4-methoxyflavanone 7-rhamnoglucoside), troxerutin (a derivative of the naturally occurring bioflavonoid rutin), and hidrosmin [3′,5-di-O-(hydroxyethyl) diosmin]. These drugs are mostly used for treating inflammation, edema, and venous insufficiency [38–40]. Naringenin (4,5,7-dihydroxy-2-(4-hydroxyphenyl)-2,3-dihydrochromen-4-one) is a flavanone that also has demonstrated biological activity with potential application in therapy.

Studies have shown that oral intake of flavonoids can reduce the harm inflicted on the glomerular filtration barrier due to hyperglycemia by hindering signaling pathways linked to kidney damage [26]. Flavonoids activate mechanisms that encompass antioxidative and anti-inflammatory properties and potential antidiabetic, antihypertensive, antifibrotic, anti-remodeling, and antiapoptotic effects. Additionally, flavonoids exhibit an antihypertensive effect by promoting diuresis and natriuresis and decreasing circulating volume, cardiac output, and vascular resistance [41, 42].

### Naringenin

Naringenin (4,5,7-dihydroxy-2-(4-hydroxyphenyl)-2,3-dihydrochromen-4-one) is a flavonoid with a molecular weight of 272.26 g/mol and belongs to the group of flavanones. It has a characteristic structure of a linear 3-carbon chain (C6-C3-C6), arranged in an oxygenated heterocyclic nucleus disposition (Figure 2) [43, 44]. In nature, naringenin is widely found in the fruit and peel extracts from the *Citrus* genus of the *Rutaceae* family in two primary forms: aglycosylated (naringenin) and glycosylated (naringin or naringenin-7-O-glycoside) (Figure 2).

Naringenin has been identified as the compound responsible for the bitter taste in the juice and peel of various citrus fruits such as lemon, orange, mandarin, and grapefruit. The concentration of naringenin in citrus fruits has been reported to range between 50 and 1200 mg/L, with the highest concentrations in grapefruit (43.5 mg/100 mL), followed by orange juice (2.13 mg/100 mL), and lemon juice (0.38 mg/100 mL) [43, 45, 46]. However, grapefruit juice as a source of naringenin should be used under strict medical surveillance because it has been reported to affect the bioavailability and effectiveness of statins, commonly used for controlling dyslipidemia in type 2 diabetes [47].

Naringenin undergoes hydrolysis in the liver facilitated by the enzyme lactase hydrolase and can subsequently undergo phase I and phase II metabolism processes involving oxidation or demethylation by cytochrome P450 monooxygenases. Subsequently, it may undergo glucuronidation, sulfation, or methylation by enzymes, such as UDP-glucuronosyltransferases (UGT) and sulfotransferases (SULT), resulting in metabolites that are excreted in the urine. Thus, naringenin and its metabolites are excreted through feces and urine [48].

As with other flavonoids, the antioxidant effects of flavanones, especially naringenin, are conferred by hydroxyl groups and a double bond in their structure. Thus the antioxidant activity is attributed to the hydroxyl groups in the 7-OH, 4′-OH, and 5-OH positions and the 4(═O) carbonyl group on the central ring (Figure 2) [44]. However, naringenin’s antioxidant activity is lower than that of other flavonoids, which has been attributed to the absence of the C2═C3 double bond [49]. Other naringenin-related activities include anti-inflammatory, antiviral, anticancer, and immunomodulatory effects [50, 51]. On the other hand, the glycosylated form of naringenin, naringin has also shown cytoprotective effects through antioxidant mechanisms [52].

### Therapeutic effects of naringenin

As described in the previous section, diabetes, through the activation of several metabolic and signaling pathways, increases ROS formation, thereby activating other inflammatory, fibrotic, and apoptotic pathways. In addition to hyperglycemia, controlling oxidative stress may be an important therapeutic target to delay the development and progression of CKD. Because most flavonoids have potential as antioxidants, naringenin has also been assessed for this biological activity in experimental models of diseases associated with oxidative stress, including diabetes.

### Effects of naringenin on hyperglycemia

Naringenin has demonstrated positive effects on diabetes in both clinical studies and experimental models. These effects include muscle, liver, adipose tissue, and pancreatic function improvements. Such effects are attributed to increased glucose uptake, insulin secretion, and improved insulin sensitivity in peripheral tissues [53–56]. In this respect, it is well known that hyperglycemia is the main target for controlling diabetic complications, including microvascular dysfunction. In diabetic rats, naringenin restored pancreatic β cell mass and improved glucose metabolism and enhanced glucose-stimulated insulin secretion in isolated rat islets in an activation Erβ-dependent way as observed in in vivo experiments [53, 56, 57]. Also, naringenin induced the expression of genes, such as estrogen receptor-a (*ERa*), fibroblast growth factor 21 (*FGF21*), pancreatic and duodenal homeobox 1 (*Pdx1*), and V-Maf musculoaponeurotic fibrosarcoma oncogene homolog A (*MafA*), which are closely linked to improved β-cell function [53, 57]. In skeletal muscle cells naringenin enhanced glucose uptake by significantly increasing AMP-activated protein kinase phosphorylation (AMPK phosphorylation/activation) [58]. Additionally, naringenin improved lipid profile (LDL-c, HDL-c, triglycerides, and total cholesterol) in an experimental model of type 2 diabetes [56].

Thus, naringenin’s effects on glucose homeostasis include improving pancreatic function, insulin secretion, and glucose uptake in peripheral tissues, which are mediated by the increase in the function and expression of glucose transporter 4 (GLUT4).

### Effects of naringenin on oxidative stress in the kidney

**Figure 3.**
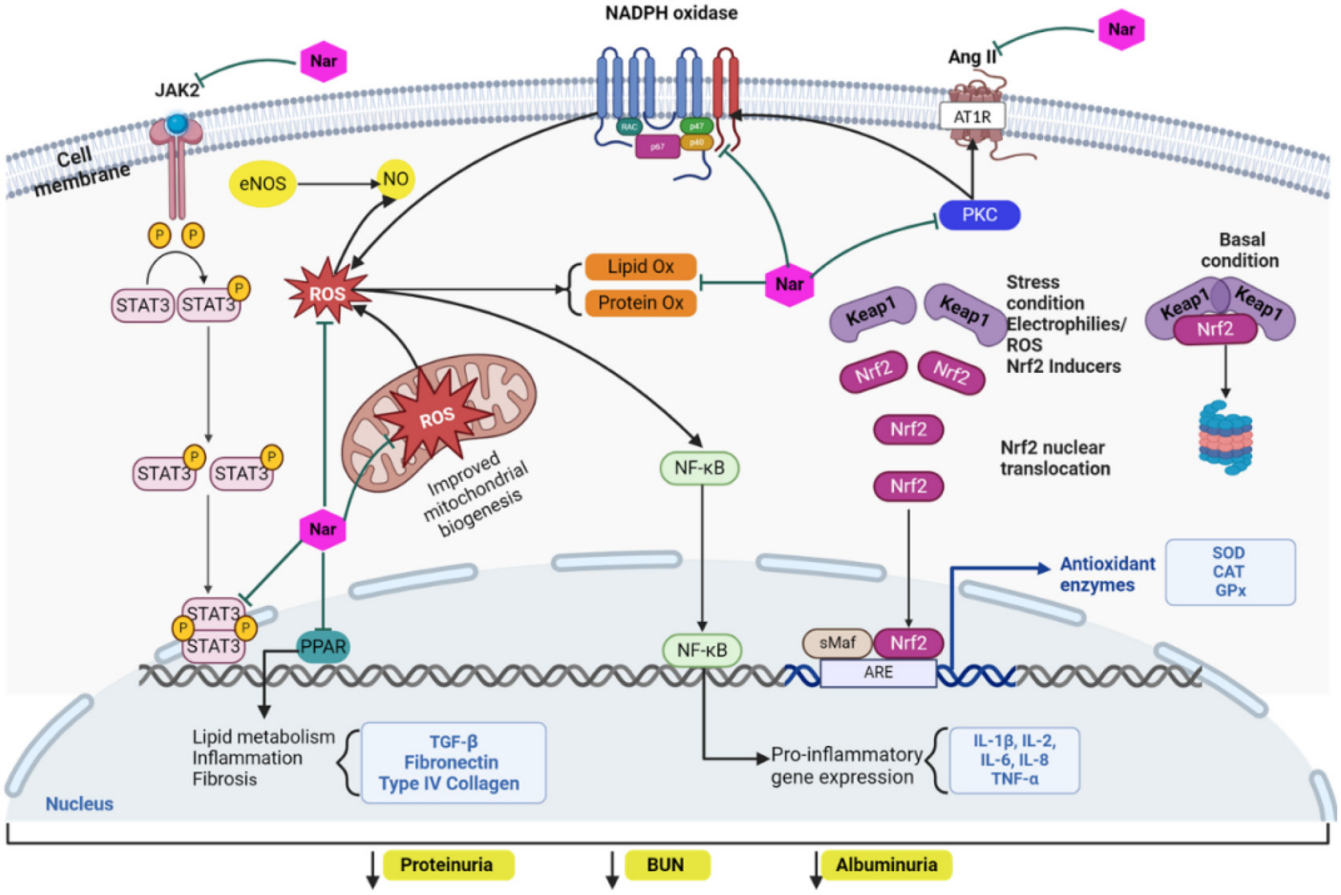
**Cell mechanism involved in the nephroprotective effects of naringenin.** Naringenin reduces oxidative stress by inhibiting the activity of enzymes that produce ROS, such as NADPH oxidase, and by improving mitochondrial function. Indirectly, through its anti-inflammatory effects and modulation of the expression of antioxidant enzymes, naringenin blocks ROS production, fibrosis, and inflammation. Ang II: Angiotensin II; ATR1: Angiotensin II type 1 receptor; ARE: Antioxidant response element; CAT: Catalase; eNOS: Endothelial NO synthase; GPX: Glutathione peroxidase; JAK2: Janus kinase 2; Keap1: Kelch-like ECH-associated protein 1; Nar: Naringenin; NO: Nitric oxide; NADPH: Nicotinamide adenine dinucleotide phosphate; Nrf2: Nuclear factor erythroid 2-related factor 2; NF-κB: Nuclear factor kappa B; PKC: Protein kinase C; PPAR: Peroxisome proliferator-activated receptors; ROS: Reactive oxygen species; TNF-α: Tumor necrosis factor-alpha; TGF-β: Transforming growth factor beta; SOD: Superoxide dismutase; STAT3: Signal transducer and activator of transcription 3.

In the kidneys of diabetic animals, naringenin treatment reduced lipid peroxidation and increased superoxide dismutase (SOD) and catalase (CAT) activities (Figure 3). Furthermore, it reduced apoptosis and the expression of TGF-β and IL-1β [59]. Regarding dyslipidemia as a contributing factor to CKD, naringenin has shown notable advantages. In *ApoE*-/- knockout mice, naringenin ameliorated dyslipidemia, atherosclerotic lesion formation, and vascular senescence. These beneficial effects of naringenin were induced by decreasing ROS formation and increasing the activities of antioxidant enzymes and the protein expressions of mitochondrial biogenesis-related genes. Naringenin treatment also enhanced the protein expression and activity of ATP synthase and sirtuin 1 (SIRT1), increasing the deacetylation and protein expression of SIRT1’s target genes forkhead box protein O3a (*FOXO3a*), and peroxisome proliferator-activated receptor a (PPARa) coactivators 1 alpha (*PGC1α*) [60, 61]. Other studies in experimental models of diabetes have reported that naringenin was able to decrease hyperglycemia, creatinine, and urea in plasma, as well as to reduce malondialdehyde (MDA), IL-1β, IL-6, TNF-α, and TGF-β levels in both plasma and the kidney. In contrast, reduced glutathione and the activities of SOD and CAT were increased in the diabetic kidney with naringenin treatment. These effects improved the histology and architecture of glomeruli and tubules and reduced apoptosis, contributing to attenuated renal dysfunction induced by diabetes including decreased hyperfiltration, microalbuminuria, polyuria, and creatinine clearance (Figure 3) [56, 62, 63].

In an experimental model of diabetes, combining naringenin treatment with an antihypertensive drug improved biochemical and urine parameters, aligning with the reduction of oxidative stress and renal damage [64]. These results suggest that naringenin alone or in combination can induce renal protection.

### Cardiovascular effects of naringenin and their role on diabetic nephropathy progression

A crucial element in renal dysfunction and CKD is hypertension, which frequently coexists with diabetes and metabolic syndrome. Hypertension is closely linked to the imbalance between vasodilatory and vasoconstrictive substances, modulation of vascular tone, and oxidative stress. In experimental hypertension, naringenin decreased blood pressure, ROS, proteinuria, plasma levels of vasodilation converting enzyme (VCE), α-1A adrenergic receptor (α-ADR) activation, and angiotensin. In contrast, naringenin increased SOD and NO levels in serum and vascular endothelial cells (Figure 3). Also, the serum levels of IL-2, IL-6, and TNF-α were decreased, while IL-10 was increased. Interestingly, naringenin inhibited Janus kinase 2/signal transducer and activator of transcription 3 (JAK2/STAT3) signaling by suppressing Src homology region 2 (SH-2) domain-containing phosphatase 1 (SHP-1) expression in vascular endothelial cells [65]. Additional protective effects of naringenin on kidney damage may stem from its capacity to regulate vascular tone by influencing enzymes responsible for metabolizing vasoactive substances and hypertensive mechanisms. In experimental models of NO-dependent hypertension, naringenin treatment reduced the expression of Ang II converting enzyme type 1 (ACE1), Ang II synthesis, oxidative stress, kidney damage, and cardiac hypertrophy. However, at the systemic level, it did not reduce blood pressure or plasma concentrations of Ang II [66, 67]. On the other hand, in a model of renovascular hypertension (2-kidney 1-clip [2K1C]), treatment with naringenin decreased plasma concentrations of Ang II and lowered the expression of ACE II in the kidney, but it increased the expression of the Ang II type II receptor (AT2R) [68]. Therefore, based on the available literature, the antihypertensive effects of naringenin through the modulation of RAAS are inconclusive, and additional investigations are required to elucidate the conflicting results.

However, other studies have explored other mechanisms involved in regulating vascular tone and renal protection. In diabetic mice, administering naringenin improved fasting blood glucose (FBG) and reduced renal damage, as demonstrated by lowering blood urea nitrogen (BUN), serum creatinine, and urinary albumin [69]. The renoprotective effects of naringenin at the structural level include the attenuation of renal tubule dilation, vacuolated lesions, mesangial expansion, thickening of GBM, renal hypertrophy, and glomerular changes [69]. At the molecular level, naringenin upregulated peroxisome proliferator-activated receptors (PPARα, PPARβ, and PPARγ) and cytochrome P450 isoform 4A (CYP4A) expressions, while the levels of 20-hydroxyeicosatetraenoic acid (20-HETE) in serum were restored [69]. Through in vitro assays, the effects of naringenin were also evaluated, confirming the observations made in animal models [69]. 20-HETE contributes to regulating kidney function, blood pressure, and vascular tone. Thus, naringenin might enhance renal function by improving kidney hemodynamics in diabetes. In an experimental model of gestational hypertension, treatment with naringenin decreased hypertension, serum markers of oxidant stress, inflammation, and serum concentrations of Ang II and ET-1, while NO and SOD were increased (Figure 3) [65]. The protective effect of naringenin in endothelial dysfunction has been further demonstrated using aortic rings from diabetic animals, preserving endothelial function and vascular reactivity via a NO-dependent mechanism [70].

Another protective effect of naringenin on the kidneys includes a decrease in proteinuria, renal and glomerular hypertrophy, along with a reduction in the expression of type IV collagen (Col IV) and fibronectin. It also modulates the TGF-β/Smad pathway both in vivo and in vitro [71]. In an experimental model of obesity-associated hypertension, naringenin treatment led to reductions in body weight and blood pressure while regulating lipid parameters by lowering total cholesterol, triglycerides, and LDL-c, and increasing HDL-c levels. Moreover, naringenin reduced serum levels of MDA, NO, and leptin, while increasing serum levels of SOD and adiponectin [72].

Also, in diabetic mice, naringenin administration reduced hyperglycemia, albuminuria, and BUN, while increasing insulin and creatinine clearance through anti-inflammatory mechanisms. Inflammation was mitigated through the modulation of TNF-α, IL-1β, IL-6, monocyte chemoattractant protein-1 (MCP-1), and NF-κB expressions in renal tissue. Furthermore, naringenin exhibited antifibrotic effects by downregulating the expression of Col IV, fibronectin, and TGF-β1 in the kidney (Figure 3) [73].

On the other hand, in the heart of diabetic animals naringenin prevented cardiac remodeling and fibrosis by reducing oxidative stress through modulation of NADPH oxidase (NADPHox) and SOD activities, as well as the regulation of the expression of protein kinase C (PKC) and p38α [74]. These effects were associated with improved FBG and reduced polydipsia and body weight loss [74].

Hyperuricemia is a prevalent chronic metabolic condition often associated with diabetes, metabolic syndrome, and hypertension, all closely linked to CKD. Naringenin has demonstrated the ability to reduce serum uric acid levels in a dose-dependent manner, likely by enhancing uric acid elimination in urine. Furthermore, naringenin reduced the expression of glucose transporter type 9 (GLUT9) by inhibiting the phosphatidylinositol 3-kinase/protein kinase B (PI3K/AKT) signaling pathway and enhanced the expression of adenosine triphosphate (ATP)-binding cassette efflux transporter G2 (ABCG2) mediated by modulation of PDZK1 abundance. The naringenin-induced uricosuric effect was associated with a decrease in IL-6 and TNF-α, which contribute to the inhibition of the TLR4/NF-κB signaling pathway [75]. Another study in an experimental model of kidney damage induced by hyperuricemia reported that naringenin reduced hyperuricemia, TNF-α, NF-κB, Cit C, and 8-OHdG, but increased glutathione peroxidase [76]. The protective effects of naringenin in hyperuricemia were also observed in liver tissue through the same mechanisms [77].

Conversely, the dysregulation of vasoactive substances and oxidative stress is pivotal in aging, a significant cardiovascular risk factor. Research conducted in an aging model revealed that naringenin safeguarded the heart against ischemia-reperfusion injury. The protective effects were mediated by the improvement in mitochondrial membrane potential, cardiac function, and reduction of myocardial infarct area [78]. Another investigation demonstrated the cardioprotective properties of naringenin in diabetes, attributed to the upregulation of CYP2J3 expression, leading to increased levels of epoxyeicosatrienoic acids (EETs), and the activation of peroxisome proliferator-activated receptors (PPARs). These mechanisms collectively contributed to the attenuation of cardiac hypertrophy [79]. Another study reported that naringenin increased SOD activity, but decreased MDA level, NOX2 expression, and MAPK signaling pathway, which improved cardiac function and decreased fibrosis and hypertrophy [80]. Cardiac hypertrophy, a form of cardiovascular disorder associated with diabetes and CKD, poses an elevated cardiovascular risk for patients, leading to conditions, such as coronary artery disease, heart failure, arrhythmias, and sudden cardiac death [81]. Hence, naringenin’s antioxidant and anti-remodeling properties could potentially provide therapeutic benefits for both the heart and kidneys.

## Discussion

Historically, traditional medicine has been utilized as an alternative therapeutic option for healing several illnesses. Evidence of therapeutic effects has been obtained from preclinical studies using experimental models and pilot studies in patients. These results indicate the presence of various compounds of natural origin with biological activity, responsible for the observed therapeutic effects [35, 82, 83]. Such is the case of naringenin, a compound that has demonstrated several cytoprotective biological activities, suggesting its potential as a therapeutic coadjuvant option for diabetes [36, 38, 39].

Oxidative stress has been described as one of the main contributors to endothelial dysfunction and hypertension during diabetes. Both diseases often coexist and increase the risk of developing micro- and macrovascular complications, including CKD and death from cardiovascular causes [84]. Further, hypertension is a prevalent comorbidity in patients with CKD and worsens with the decline in renal function. Therefore, therapeutic strategies for diabetes must be focused on controlling oxidative stress, endothelial dysfunction, inflammation, and hypertension, in addition to strict blood glucose control, to contribute overall to slowing the progression of CKD [32, 85, 86].

To this respect, naringenin has demonstrated antioxidant effects by directly acting as a ROS scavenger [87, 88], and indirectly through the inhibition of the activity of enzymes that produce ROS, including NADPH oxidase and myeloperoxidase (MPO) [60, 65, 88]. It also modulates the expression of the antioxidant enzymes including SOD, and CAT (Figure 3) [59, 60, 74]. Another antioxidant effect of naringenin may be indirectly mediated by its immunomodulatory effects (Figure 3) [54, 65, 73, 75, 89]. These effects were observed on TNF-α, CD68, and IL-1β, and were mediated through the regulation of phosphorylation of NF-κB p65, JNK, and ERK related to inflammation, as well as Bcl2, Bax, p53, apoptotic factor poly (ADP-ribose) polymerase 1 (c-PARP), and caspases 3, 8, and 9, which are linked to apoptotic signaling in the pancreas [53].

Naringenin’s anti-inflammatory properties might offer therapeutic potential by mitigating oxidative stress and disrupting the detrimental cycle of inflammation and oxidative stress, which contribute to kidney dysfunction and CKD [85, 86, 90, 91].

Another indication of naringenin’s potential as a beneficial treatment option for diabetic nephropathy is its ability to modulate vascular tone and the synthesis of vasoactive substances. These include NO, Ang II, endothelin, EETs, and 20-HETEs [65–69]. Equally significant are its effects on mechanisms associated with tissue remodeling, such as reducing the expression of TGF-β, Col IV, and fibronectin [71, 73].

As of now, there have been no interventional studies conducted using naringenin for the treatment of diabetic nephropathy. However, promising outcomes have been observed in other conditions. For instance, naringenin inhibited tumor growth in osteosarcoma patients and decreased the recurrence rate. It also led to reductions in IL-1β and IL-6 levels, as well as ROS levels compared to the placebo group, while elevating SOD and glutathione (GSH) levels in plasma in a time-dependent manner [92].

There is some understanding regarding the safe doses of naringenin for human consumption. In a single-ascending-dose randomized crossover trial assessing naringenin’s safety, it was reported that doses ranging from 150 to 900 mg/day are safe for healthy adults, with serum concentrations showing proportionality to the administered dose. A dose of naringenin at 8 µM was found to be effective in primary human adipocytes. Ingesting 300 mg of naringenin twice daily will likely produce a physiological effect [93]. Once administered, naringenin is absorbed in the gastrointestinal tract and subsequently distributed in the blood, lungs, trachea, liver, and kidneys, although its bioavailability is approximately 15% [94, 95]. The metabolism of naringenin is carried out in the liver through processes of oxidation, demethylation, glucuronidation, sulfation, or methylation and it is finally excreted by the kidney and eliminated in urine [96].

Variations in the effects of naringenin could stem from differences across trials, including variations in the source, purity, vehicle, doses, and duration of intervention. Moreover, the absence of standardized protocols for administration further complicates interpretation. On the other hand, the consumption of fruit juice as a source of naringenin should be avoided because other compounds in the juice can interfere with the activity of the drugs and cause side effects, such as with the success of statins [47]. The limitations of this work include a lack of in-depth exploration of the side effects on target organs and the underlying cellular mechanisms. This is due to the primary focus of this review being on highlighting the beneficial effects of naringenin and promoting its potential as a therapeutic option. Regrettably, current interventions to treat diabetic nephropathy have shown limited success, at best delaying disease progression to its advanced stages. Consequently, there is an urgent need for novel preventive or efficacious options. Recently, other therapeutic targets in diabetes have emerged: the preservation of endothelial function and the management of dyslipidemia, oxidative stress, inflammation, and fibrosis.

## Conclusion

Through direct mechanisms and the modulation of signaling pathways, naringenin exhibits beneficial effects that include antihyperglycemic, antioxidant, anti-inflammatory, anti-remodeling, and antihypertensive properties. Experimental models of renal damage have demonstrated that naringenin protects endothelial and renal function, thus slowing the progression of renal disease. Additionally, there have been no reports of adverse effects in humans. Therefore, naringenin shows promise as a therapeutic option for diabetes-induced comorbidities. Nonetheless, further clinical studies are required to validate its efficacy as either a primary or adjunctive medication.
